# KEEPERS OF CONTEXT: CENTERING VOICES OF CONCERNED PERSONS IN THE LIVES OF OLDER ADULT VICTIMS

**DOI:** 10.1093/geroni/igae098.0578

**Published:** 2024-12-31

**Authors:** Lisa Rachmuth

**Affiliations:** Weill Cornell Medicine, New York, New York, United States

## Abstract

Older adults who experience financial exploitation and other forms of abuse often experience this abuse in isolation. However, financial exploitation and other forms of abuse can also profoundly impact another group of individuals in the lives of older adults: non-abusing family members, friends, and neighbors, referred to as “Concerned Persons” (CPs). The Concerned Person in the life of the older adult is often one of the few persons that can help identify the abuse and find resources to help the older adult move out of the abuse. Concerned Persons in the lives of older adults are sometimes secondary victims of the abuse, isolating themselves from other support networks and at times putting themselves at risk in various ways. A series of focus group conducted in 2024 focused on factors that motivated Concerned Persons in the lives of the older adult to help, how Concerned Persons can help professionals understand the older adults’ experiences with abuse across the lifespan, and how they can affect change for the older adult. Focus groups were conducted in affiliation with the Elder Abuse Helpline for Concerned Persons (the Helpline), a program at Weill Cornell Medicine. Through the Helpline, CPs are able to receive supportive counseling for themselves and referrals to ongoing services for themselves and the Older Adults who are abused. Findings from this research surface new insights that connect Concerned Persons’ experiences and attitudes with the topic of abuse across the lifespan, pointing to implications for research, policy, and practice.

